# Developing an Anti-Racist Foundations Course in MCH for MPH Students

**DOI:** 10.1007/s10995-021-03285-2

**Published:** 2022-01-04

**Authors:** Cassondra Marshall, Michael Bakal, Julianna Deardorff, Cheri Pies, Michael C. Lu

**Affiliations:** 1 School of Public Health, University of California, 2121 Berkeley Way #5302, BerkeleyBerkeley, CA 94720 USA; 2 Graduate School of Education, University of California, 2121 Berkeley Way 4th Floor, BerkeleyBerkeley, CA 94720 USA

**Keywords:** Racism, Anti-racist pedagogy, Graduate education, MCH education

## Abstract

**Introduction:**

Over the past decade, foundational courses in MCH have been revised and revamped to integrate the life course perspective and social determinants of health in ways that bring these essential issues to the core of the learning experience. Yet the racial reckoning of 2020 and the racially disparate health impacts of the COVID-19 pandemic underscore that a deeper, more focused approach to anti-racist pedagogy is now imperative for MCH educators and others responsible for developing the MCH workforce.

**Methods:**

In this paper, we discuss our experience of building a ‘community of practice’ of anti-racist MCH trainees through our course, ‘Foundations of Maternal and Child Health Policy, Practice, and Science.’

**Results:**

We identify four principles which guided our course: (1) building on students’ experience, knowledge, identities and social justice commitments; (2) creating a common purpose and shared vocabulary related to racism; (3) organizing classroom activities to reflect real-world problems and professional practices related to addressing structural racism as a root cause of health inequities; and (4) building students’ skills and confidence to recognize and address structural racism as MCH professionals.

**Discussion:**

We hope that this description of our principles, along with examples of how they were put into practice, will be useful to MCH educators who seek to build anti-racist frameworks to guide MCH workforce development.

## Significance

*What is already known on this subject?* The racial reckoning of 2020 and the racially disparate health impacts of the COVID-19 pandemic underscore that a deeper, more focused approach to anti-racist pedagogy is now imperative for MCH educators and others responsible for developing the MCH workforce.

*What this study adds?* We discuss our experience of building a ‘community of practice’ of anti-racist MCH trainees through our foundational MCH course. We describe four design principles, along with specific practices. Our goal is to provide an example for others who seek to build anti-racist frameworks to guide MCH workforce development.

## Introduction

During Summer 2020, the United States was in the midst of two crises, with racial inequities front and center of both. George Floyd’s horrific murder in May 2020 prompted a national reckoning on structural racism and police violence. At the same time, national public health data illustrated that the unrelenting toll of COVID-19—as many predicted—was greatest among Black, Indigenous, and people of color (BIPOC) (Eligon et al., 2020).

As we (authors CM and MB) prepared to teach our Fall course, ‘Foundations of Maternal and Child Health Policy, Practice, and Science’ (Foundations), we had a clear understanding that our curriculum and pedagogy needed to evolve to meet the moment. We felt a strong moral responsibility to make our class responsive to the structural racism that was playing out in front of everyone’s eyes (Krieger, 2020), and to prepare students to understand and embrace what it means to be anti-racist MCH leaders. At the same time, Michael Lu, dean at the School of Public Health at UC Berkeley School of Public Health, issued a challenge for the School to become more anti-racist, which included incorporating anti-racist content and pedagogy throughout the School’s curriculum, and ensuring that inclusion and belonging were consistently cultivated in all classroom environments.

As a first step, we set out to better equip our MCH trainees to understand, recognize, and confront racism through our Foundations course. The Foundations course introduces MPH students to key MCH topics, including the history and organization of MCH services and programs, MCH policy, the life course perspective, and key issues for MCH populations (e.g., mothers, adolescents, children with special health care needs, etc.). The course is designed to lay a foundation for students’ training throughout the MPH program and impart key skills necessary to build a dynamic and effective MCH workforce.

Given stark and persistent disparities by race across numerous MCH outcomes, racial health disparities have long been a focus of Foundations. For example, Black mothers, across the income and educational spectrum, are three to four times more likely to die from pregnancy-related complications than their non-Hispanic white counterparts (Artiga et al., 2020). Infants born to Black, Native Hawaiian and Pacific Islander, American Indian and Alaskan Native mothers are about twice as likely to die before age 1 as infants born to white mothers (Artiga et al., 2020). Further, amidst growing recognition that racism is a public health crisis (American Public Health Association, 2020; Ford, 2019), children’s health professionals and advocates have been called upon to develop policies and strategies that adopt a ‘racism as a root cause’ approach (Malawa et al., 2021). This directive points to a need to develop an MCH workforce with the knowledge, skills, and commitment to confront structural racism.

Thus, while our Foundations course already had a strong emphasis on the life course perspective, social determinants of health, and social justice, we believed that the moment demanded that we redouble our commitment to equip the next generation of MCH leaders to dismantle structural racism. Below we discuss key elements of the design of our Foundations course as a community of practice aimed at training an anti-racist MCH workforce. We hope that this description of our principles, along with the specific practices we implemented, provides a useful framework for greater consideration of anti-racist workforce development in MCH. We focus not on the MCH content of the course but rather on our approach to developing an anti-racist pedagogy and class community. We note that the incorporation of anti-racist pedagogy and teaching practices are receiving increased attention in response to current events (Landau, 2021; McMurtrie, 2020), and, as such, we have provided concrete examples that can be adapted for courses in public health more broadly and in other disciplines.

## Community of Practice (COP)

Communities of practice are “…groups of people who share a concern or a passion for something they do and learn how to do it better as they interact regularly” (National Network of Public Health Institutes, 2020; Wenger-Trayner & Wenger-Trayner, 2015). The concept emerged from ethnographic studies of how learning takes place in informal settings, such as apprenticeships. In such contexts, learning frequently takes place as newcomers participate and assist in carrying out authentic tasks in real-world settings. Rather than engage in training exercises in specialized sites of learning, learning takes place through observing and “pitching in” with master practitioners (Lave & Wenger, 1991; Rogoff, 2003).

In MCH and public health more broadly, the communities of practice approach is widely used in the professional development of already-trained public health practitioners (Association of Maternal & Child Health Programs, n.d.; Barbour et al., 2018; Meagher-Stewart et al., 2012). The National Network of Public Health Institutes (2020) finds this approach consistent with the requirements of the public health profession, writing that communities of practice are “an effective strategy to create, disseminate and implement new knowledge to address the complex health issues of our time” (p.4).

In the context of Foundations, our rationale for organizing the class as a community of practice as a means to equip students to challenge structural racism was two-fold: First, we believed this approach would help create a sense of community and shared purpose, which we knew would be indispensable for building the skills and fostering the trust necessary to engage in difficult conversations about structural racism. This sense of community and shared purpose, in turn, would equip students with the courage and community to challenge structural racism as MCH professionals. Second, we believed it would help our students *see themselves as transformative leaders in the field of MCH.* As research shows, when learners positively identify with a profession or academic domain, they are deeply motivated to master its relevant skills and concepts (Osborne & Jones, 2011; Sfard & Prusak, 2005).

## Building an Anti-Racist MCH Community of Practice

While we embarked on our Fall 2020 semester with a clear goal of redoubling our commitment to anti-racist pedagogy, our objectives and classroom practices only began to crystallize during the process of teaching the course – we were truly “building the plane while flying it.” Furthermore, only upon reflection were we able to distill our teaching into the following set of four design principles and accompanying classroom practices (Table 1). Below, we describe each principle and elaborate on how it was integrated into practice.

**Table 1:** Design Principles and Practices for our MCH Foundations Course

Design Principles	Classroom Practices
Build on students’ experience, knowledge, identities & social justice commitments	●Public health autobiographies ●Reflection on positionality and its relevance to MCH issue ●Ongoing opportunities to relate new content to students’ lives and experiences
Create a common purpose & shared vocabulary around racism and health equity in MCH	●Readings, videos, and podcasts focused on racism and MCH ●Deep learning and application of conceptual frameworks ●Dynamic and shared reading annotations
Organize classroom activities to reflect real-world MCH problems and professional practices related to addressing racism as a root cause of health inequities	●Highlighting local, community driven MCH efforts and interventions that are tackling ‘racism as a root cause’ through guest speakers
Build students’ skills and confidence to identify, address, and respond to structural racism as MCH professionals	●Students’ final projects—including a problem statement, policy brief, and TED-style talk—are of professional standards and equip them to dismantle racist systems

### Build on Students’ Experience, Knowledge, Identities, and Social Justice Commitments

Our experience teaching Foundations showed us that our students entered the program with an existing wealth of prior experiences—from their homes, communities, and professional lives—which together constituted a rich foundation for learning in MCH. Luis Moll (1998) refers to the knowledge learners bring from their homes, communities and cultures as “funds of knowledge,” which are a vital but often untapped resource for learning. Consistent with the funds of knowledge approach, our aim was to elicit and leverage students’ lived experiences, background knowledge, and professional experiences to facilitate both the learning of new content and students’ learning from each other. Valuing students’ identities and prior experiences helped create a humanizing pedagogy (Salazar, 2013) and syncretic curriculum (i.e. one which blended learners’ everyday and experiential knowledge with foundational MCH concepts) (Gutiérrez, 2008), and showed that we, as educators, were as eager to learn from students as we hoped they would be to learn from us.

To help students feel seen and welcomed, their first assignment was to complete a “public health autobiography” which responded to the prompts: (1) What was my road to public health? What brought me to an MCH course? and (2) What experiences and/or people in my life shaped me and motivated my interest in public health and MCH? Students shared their autobiographies with one another and we encouraged them to make connections with one another around shared interests and experiences.

Later in the course, students recorded short self-reflection videos in which each student responded to the prompt: "How is your MCH topic of interest influenced by your personal or professional experiences and your positionality (i.e. your experience of race, class, gender identity and expression, religion, etc.)?" Students then watched the reflection videos of their classmates. Our goal in encouraging students to reflect on their positionality was to signal that their identities, experiences, and commitments were important assets to the collective work we would be doing in the course and beyond. This exercise also provided an opportunity for students to engage with their own privilege and power, a key tenet of social justice pedagogy, and to use this as a lens through which to view MCH research and practice (Taylor et al., 2019).

### Create a Common Purpose and Shared Vocabulary Related to Addressing Racism as a Root Cause of Health Inequities

We designed our class mindful of several related considerations. We knew that many of our students and faculty were part of communities that directly experience racism’s harmful effects, and thus for whom these issues are personal, often acutely so. Given this understanding, we felt it was important to provide time to collectively process the emotional weight of the health inequities we were studying and to create space for students to bring experiential knowledge into our conversations. We were also aware that some students entered the program with less understanding of the impact of racism and inequities on health, but were eager to learn. We thus co-created, with students, a working set of community agreements which emphasized a commitment to learn from one another and openness to change and extend our perspectives.

Another important consideration was to help students not only understand racism and the ways in which racism influences health, but also to be effective and thoughtful communicators about racism and health — both in our class conversations and with broader audiences. To this end, we knew that a shared set of conceptual frameworks and vocabulary would be essential. We built this shared vocabulary and conceptual foundation in three main ways. First, students were assigned several texts,^1^ including peer-reviewed journal articles, webinars from professional associations, and a journalistic podcast, that specifically focused on the relevance of racism to MCH issues. Second, we introduced a set of guiding theoretical and conceptual frameworks that would be referenced continually throughout the course. These frameworks were: the life course perspective (Lu & Halfon, 2003), the socioecological model (Alio et al., 2010), reproductive justice (Ross & Solinger, 2017), and health equity (Braveman, 2014). These frameworks were introduced through readings and group discussions which were student-led, with guidance from instructors. Third, we ensured these theories would serve as ongoing points of reference throughout the course. To this end, students worked on interactive reading annotations in which they were required to annotate and comment on weekly readings which were uploaded as a virtual document and shared among class members (Cortez et al., 2021). As students completed each weekly reading, they left comments which were visible to the others, in response to passages from the text and to other classmates’ comments. This task created a rich and dynamic opportunity for students to engage in asynchronous dialogue, which was particularly helpful for overcoming some of the challenges of online learning.

Throughout the course, students’ reading annotations reflected ongoing engagement with issues of racism, the life course perspective, and health equity. For example, in many reading annotations and class discussions, students posed questions and offered comments that drew our collective attention to communities whose needs may not always be adequately met by the field of MCH, such as BIPOC trans communities. These comments helped us engage more deeply with intersectionality (Bowleg, 2012), and how we can build upon and extend existing MCH frameworks and programs to better serve all communities.

### Organize Classroom Activities to Reflect Real-World Problems and Professional Practices Related to Addressing Structural Racism as a Root Cause of Health Inequities

Consistent with the Communities of Practice approach, a key task of training the future MCH workforce was to help students become part of broad networks of MCH professionals working to push the envelope in our field in their anti-racist practice. To achieve this goal, we introduced students to individuals and programs engaged in transformative, anti-racist public health research, policy development and practice. Throughout the semester, we sought to include guest speakers with whom students could identify and even “see themselves becoming in the future.” This process began with prioritizing racial and gender diversity in our selection of speakers. Yet beyond mere representation, our foremost concern was to inspire students by providing them with opportunities to engage with bold transformative leaders in the field who had a guiding concern with addressing questions of equity and/or structural racism. Our speakers worked in a variety of settings, including MCH departments, academic institutions, and community-based programs. To offer one example, each year we host a speaker from a program based in a nearby metropolitan area implementing the ‘racism as a root cause’ approach to address vulnerabilities faced by Black and Pacific Islander women during pregnancy and childbirth, using a collective impact model (Expecting Justice, n.d.). These ongoing opportunities to interact with MCH leaders not only inspire students to develop transformative public health proposals themselves (described in the next section) but also integrate them within MCH communities of practice, thereby deepening their identification with the field.

### Build Students’ Skills and Confidence to Recognize and Address Structural Racism as MCH Professionals

Students’ final projects represented the culmination of the three previously described design principles. Picking up the thread of the issues identified in their public health autobiographies, students now were challenged to develop professional-quality deliverables about these issues. Each deliverable was required to be strongly evidence-based and grounded in the theoretical and conceptual frameworks introduced earlier in the course (described above).

Students’ final project involved three core learning tasks designed to reflect real-world products used by MCH professionals. First, students wrote problem statements in which they defined their issue of focus and delineated its public health importance. Second, students wrote policy briefs which outlined innovative policy solutions and identified relevant target audiences. Third, students delivered presentations modeled on TED Talks in which they discussed their issue and policy as they would to a general audience. These three assignments built sequentially, and students were given extensive time to meet with peer work groups and with instructors to receive guidance and feedback.

Structuring assignments to be connected to students’ values, to reflect authentic tasks of the MCH workforce, and to be collaboratively developed over time was consistent with our communities of practice approach, and we saw it fostered students’ intrinsic motivation to excel in our course. We believed students (and instructors!) were enriched by having the opportunity to learn from the wide range of MCH issues and innovative solutions explored by their peers.

## Conclusion

Growing a diverse MCH workforce that is equipped to address structural racism is imperative. In this paper, we have outlined one pedagogical approach used in a graduate-level introductory MCH course. We believe the course is dynamic, and we will continue to improve our anti-racist pedagogy as we learn and reflect as educators. We also acknowledge that a single class is not enough to build an anti-racist MCH workforce and that there is much to do, but our hope is that the principles and practices we have shared provoke a more robust conversation about how we train the next generation of MCH professionals to understand, recognize, and confront racism. We note that what we have outlined here reflects the perspective of the instructors. Moving forward, it will be important to include and evaluate students’ perspectives as curriculum and syllabi changes are made. Their perspectives will be particularly important in understanding the extent to which changes are having their intended impact.

## Data Availability

N/A.

## Footnotes

1 Examples include the March of Dimes webinar ‘Confronting Hard Truths: How Systemic Racism Affects Moms and Babies’, the Reveal podcast ‘Reproducing Racism’, and ‘The Impact of Racism on Child and Adolescent Health’ by Trent and colleagues published in *Pediatrics* (2019).

## Code availability

N/A.
